# Thrombocytopenia as a Diagnostic Marker for Malaria in Patients with Acute Febrile Illness

**DOI:** 10.1155/2021/5585272

**Published:** 2021-04-10

**Authors:** Angesom Gebreweld, Yonas Erkihun, Daniel Getacher Feleke, Gebru Hailu, Temesgen Fiseha

**Affiliations:** ^1^Department of Medical Laboratory Sciences, College of Health Sciences, Mekelle University, Mekelle, Ethiopia; ^2^Department of Medical Laboratory Sciences, College of Medicine and Health Science, Wollo University, Dessie, Ethiopia; ^3^Department of Microbiology, Immunology and Parasitology, College of Health Sciences, Addis Ababa University, Addis Ababa, Ethiopia; ^4^Department of Environmental Health and Behavioral Science, School of Public Health, College of Health Sciences, Mekelle University, Mekelle, Ethiopia

## Abstract

**Background:**

Thrombocytopenia is the most common hematological abnormality in patients with acute malaria. This study aimed to determine the role of thrombocytopenia as a diagnostic marker for malaria in patients with acute febrile illness.

**Method:**

A cross-sectional health facility-based study was conducted on 423 consecutively selected acute febrile patients at Ataye District Hospital from February to May 2019 GC. A complete blood count and malaria microscopy were performed for each acute febrile patient. ROC curve analysis was performed to calculate sensitivity, specificity, positive predictive value, and negative predictive value of platelet count in predicting malaria. A *P* ≤ 0.05 was considered statistically significant.

**Result:**

Out of the 423 acute febrile patients, 73 (17.3%) were microscopically confirmed malaria cases and the rest 350 (82.7%) patients had negative blood film results. Of the microscopically confirmed malaria cases, 55 (75.34%) were *P. vivax* and 18 (24.66%) were *P. falciparum*. The prevalence of thrombocytopenia among malaria patients (79.5%) was significantly higher than those in malaria negative acute febrile patients (13.7%), *P* < 0.001. About 67% malaria-infected patients had mild to moderate thrombocytopenia and 12.3% had severe thrombocytopenia. The ROC analysis demonstrated platelet counts <150,000/*μ*l as an optimal cutoff value with 0.893 area under the curve, 79.5% sensitivity, 86.3% specificity, 95.3% negative predictive value, and 54.7% positive predictive value to predict malaria.

**Conclusion:**

Malaria is still among the major public health problems in the country. Thrombocytopenia is a very good discriminatory test for the presence or absence of malaria with 79.5% sensitivity and 86.3% specificity. Therefore, this may be used in addition to the clinical and microscopic parameters to heighten the suspicion of malaria.

## 1. Background

Malaria is a life-threatening infectious disease caused by protozoan parasites of the *Plasmodium* species. Globally, there were an estimated 228 million malaria cases and 405, 000 deaths in 2018, and 93% of the malaria cases and 94% of the deaths were in the African Region [1, 2]. The sub-Saharan Africa region was the most affected area contributing the largest burden of malaria morbidity and mortality. In Ethiopia, malaria is a serious public health problem with around 68% of the population is at risk of malaria. Approximately, 2.9 million malaria cases and 4,782 deaths were reported in 2016. The most dominant plasmodium species are *Plasmodium falciparum* and *Plasmodium vivax* [3–5].

Malaria parasite is a blood parasite that causes different hematological abnormalities such as anemia, thrombocytopenia, lymphopenia, monocytosis, eosinopenia, and rarely disseminated intravascular coagulation [6–9]. Those hematological alterations may vary with level of malaria endemicity, background hemoglobinopathy, nutritional status, demographic factors, and malaria immunity [10]. Thrombocytopenia is the most common hematological abnormality in patients with acute malaria [9, 11]. Severe thrombocytopenia is associated with increased risk of mortality in both child and adult patients with *P. falciparum* and *P. vivax* infections [12]. The mechanism of thrombocytopenia in malaria is not clear, but the speculated mechanisms are an increase in the consumption or destruction of platelets, or suppression of thrombopoiesis, or a combination of both. Increased consumption or destruction of platelets during malarial infection occurs due to antibody-mediated platelet destruction, disseminated intravascular coagulation (DIC), pooling within the reticuloendothelial system, sequestration in the microcirculation, oxidative stress, and malaria-mediated apoptosis [13–16].

There are some studies that reported the presence of thrombocytopenia is a predictor of acute malaria in patients with acute febrile illness in endemic areas [17–20]. However, there is scarce information on usefulness of thrombocytopenia in predicting acute malaria in Ethiopia. Therefore, this study is aimed to determine the role of thrombocytopenia as a diagnostic marker for malaria in patients with acute febrile illness in Ethiopia.

## 2. Methods

### 2.1. Study Design, Area, and Population

A cross-sectional health facility-based study was conducted at Ataye District Hospital from February to May 2019 GC. The Hospital is found in Ataye Town, Ataye District, North Shewa Zone of the Amhara Region, Ethiopia. Ataye Town is located at an elevation of 1468 meters above sea level and about 270 km away from the capital city Addis Ababa. Ataye District is one of the hot and malaria endemic areas in Amhara Region, Ethiopia. Malaria is one of the major health problems of the district. Among the plasmodium species, *P. vivax* and *P. falciparum* are main plasmodium species responsible for malaria infection in the district.

A total of 423 consecutively selected acute febrile patients were enrolled from Ataye District Hospital. Acute febrile patients with pregnancy, HIV, known hematological malignancies, bleeding disorders, and antimalarial therapy were excluded from the study.

### 2.2. Data Collection Procedure

Semistructured questionnaires were used to collect sociodemographic and clinical data of the study participants by trained clinical nurses. A complete blood count (CBC) and malaria microscopy were performed for each acute febrile patient. Sysmex KX-21N (Sysmex Corporation, Kobe, Japan) automated hematological analyzer was used to determine the complete blood count (total white blood cell count, red blood cell, and platelet count). Giemsa-stained thin and thick blood smears were used to diagnose malaria. Thin smears were considered positive for malaria if one or more malarial parasites were seen and negative if no asexual form of *Plasmodium* was detected in a minimum of 200 oil immersion fields. On thick blood smears, detection of any levels of asexual forms of malarial parasites was considered malaria-positive and if no parasites were seen after examining 1000 white blood cells labeled as negative. The blood smear examination and complete blood count were performed by two experienced medical laboratory scientists. These laboratory professionals had trainings on malaria microscopy. Standard operating procedures (SOPs) were strictly followed in each step to maintain the quality of the laboratory results.

Thrombocytopenia was defined as a total platelet count <150 × 10^3^/*μ*L. Thrombocytopenia was further classified into mild thrombocytopenia (platelet counts from 100 to 150 × 10^3^/*µ*L), moderate thrombocytopenia (platelet counts from 50 to 100 × 10^3^/*µ*L), and severe thrombocytopenia (platelet counts less than 50 × 10^3^/*µ*L) [21].

### 2.3. Data Analysis

The data were entered and analyzed using a Statistical Package for the Social Science (SPSS) Version 20 statistical software. We used the *t*-test for continuous variables and the chi-square test for categorical variables to compare means and proportions. ROC curve analysis was performed to calculate sensitivity, specificity, positive predictive value, and negative predictive value of platelet count in predicting malaria. In all cases, a *P* value less than 0.05 was considered statistically significant.

### 2.4. Ethical Considerations

Ethical approval was obtained from the Research and Ethics Review Committee of College of Medicine and Health Sciences, Wollo University. Permission to conduct the study was also obtained from Ataye District Hospital. Written informed consent from adult participants and assent for children under 18 years of age were obtained before enrolment into the study. To ensure confidentiality, participants' data were linked to a code number. Any abnormal test results of participants were communicated to the concerned body in the hospital.

## 3. Result

### 3.1. Sociodemographic and Clinical Characteristics of Participants

A total of 423 subjects with acute febrile illness were enrolled in this study. The median age of the study participants was 25 years (interquartile range: 18–34), and most of the study participants (*n* = 143; 33.8%) were within 15 to 24 years of age. More than half of the study participants were females (54.8%) and rural residents (53%). About 24.8% of participants were illiterate and 25.8% were housewives. Thrombocytopenia was noted in 106 (25.1%) acute febrile patients. Fifty-four (12.8%) patients had mild thrombocytopenia, 43 (10.2%) had moderate thrombocytopenia, and 9 (2.1%) had severe thrombocytopenia (Table 1).

### 3.2. Malaria and Thrombocytopenia

Out of the 423 acute febrile patients, 73 (17.3%) were microscopically confirmed malaria cases and 350 (82.7%) had negative blood films (Table 1). Of the microscopically confirmed malaria cases, 55 (75.34%) were *P. vivax* positive and 18 (24.66%) were *P. falciparum*. There was no statistically significant difference in malaria infection between male and female acute febrile patients (14.7% and 19.4%, respectively). Malaria infection was more prevalent in rural residents (22.8%) than urban residents (11.1%), (*P* = 0.002).

The mean platelet count was significantly lower in patients with malaria infection than patients without malaria infection (*P* < 0.001). The prevalence of thrombocytopenia among malaria patients (79.5%) was significantly higher than those of malaria negative acute febrile patients (13.7%), *P* < 0.001. Regarding to the severity of thrombocytopenia, about 67% malaria-infected patients had mild to moderate thrombocytopenia and 12.3% had severe thrombocytopenia (Table 2).

**Figure 1:**
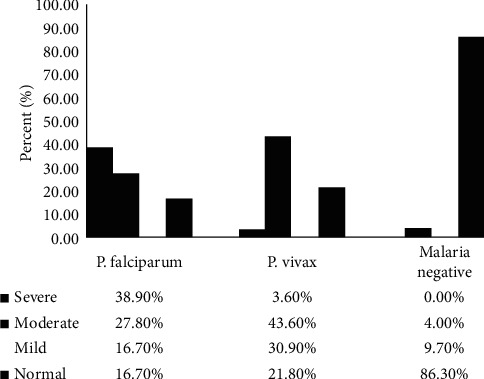
Severity of thrombocytopenia by malaria species in acute febrile patients.

The prevalence of severe thrombocytopenia was higher in *P. falciparum* (38.9%) than in *P. vivax* (3.6%) infected study participants. Most of the malaria negative acute febrile patients (86.3%) had normal platelet count (Figure 1).

### 3.3. Diagnostic Values of Platelet Count

**Figure 2:**
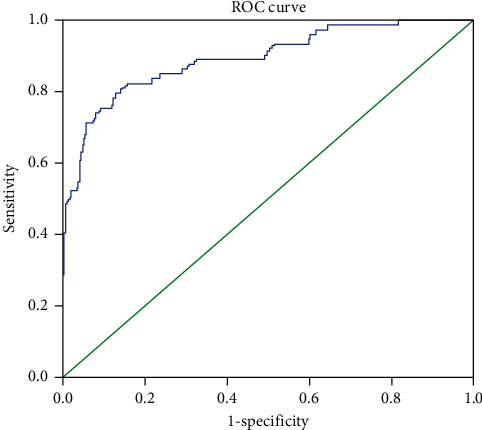
Receiver operating characteristic (ROC) curve for platelet count in acute febrile patients.

In the ROC analysis, the area under the curve (AUC) of the platelet count was 0.893 (95% CI: 0.847–0.938; *P* < 0.001), the optimum cutoff point of platelet count to differentiate malaria infection from acute febrile patients was less than 150 × 10^3^/*µ*l with sensitivity of 79.5% and specificity of 86.3% (Figure 2 and Table 3).

## 4. Discussion

Malaria is one of the high burden diseases in developing countries. Ethiopia is one of the sub-Saharan countries highly endemic to malaria where an estimated 68% of the population lives in malarious areas [4, 5]. In the present study, the prevalence of microscopically confirmed malaria cases was 17.3% which is similar to studies conducted in the Zeway Health Center, Ethiopia (17%) [22], Gurage Zone, Southern Ethiopia (18.3%) [23], East Nile locality of Khartoum State (18.5%) [24], and Kenya (15.5%) [25]. However, the finding of this study is lower than studies conducted in Arba Minch, Southern Ethiopia (27.6%) [20], Kersa Woreda, Ethiopia (43.8%) [26], Zaria, Nigeria (45.4%) [27], and New Delhi (24%) [18] and higher than studies conducted in North Shoa, Ethiopia (8.4%), and District Dir Lower, Pakistan (12.2%) [28, 29]. The observed variation might be due to seasonal climatic condition and altitude difference that might influence breeding of malaria vector and community awareness about malaria transmission and control. Among the confirmed malaria cases, the predominant *Plasmodium* species was P. vivax (75%), followed by *P.falciparum* (25%). This was in agreement with other studies [23, 29, 30], but other studies reported that the most prevalent species was *P. falciparum* [26, 28].

In this study, mean platelet counts were significantly reduced in malaria-infected patients than those with nonmalaria. Thrombocytopenia occurred in 79.5% of malaria-infected patients. These findings imply that thrombocytopenia may be a marker of *Plasmodium infection*. The association of thrombocytopenia and malaria infection was in agreement with previous studies [9, 20, 31–33]. No statistically significant difference in the prevalence of thrombocytopenia was observed between *P. falciparum* (83.3%) and *P. vivax* (78.2%) infected patients (*P* = 0.7482) which is similar to a study done by Kassa et al. [34]. In contrast to our study, Patel et al. showed significantly higher incidence of thrombocytopenia in *P. falciparum* than *P. vivax* [11] and Shaikh et al. showed significantly higher incidence of thrombocytopenia in *P. vivax* infected patients [33]. Thrombocytopenia in malaria infection might occur due to an increase in the consumption or destruction of platelets, or suppression of thrombopoiesis, or a combination of both. The suggested mechanism of accelerated clearance or consumption of platelets during malarial infection includes disseminated intravascular coagulation (DIC), immune-mediated destruction, pooling within the reticuloendothelial system, sequestration in the microcirculation, and malaria-mediated apoptosis [13–16]. In contrast to this study, a study was reported no association between the mean platelet count and irritable bowel syndrome [35]. A study was reported no difference between mean platelet count and TSH levels between the malignant thyroid nodule group and benign nodule group [36]. There are a lot of possible reasons for the increase or decrease of the peripheral platelet count. Among the different variables that can determine the mean peripheral platelet count, platelet production rate, mean platelet survival/life span, and the size of the exchangeable splenic platelet pool can be mentioned [37]. In contrast to the relationship between malaria and thrombocytopenia in the present study, a study reported by Sincer et al. showed that there was an inverse relationship between the number of blood eosinophil count and the severity of acute coronary syndrome (ACS) in elderly patients [38].

In this study, the ROC analysis demonstrated platelet counts <150,000/*μ*l as an optimal cutoff value with 79.5% sensitivity, 86.3% specificity, 95.3% negative predictive value, and 54.7% positive predictive value. The area under the curve of the platelet count was 0.893 which means thrombocytopenia is a good discriminatory test for the presence or absence of malaria. These values are very close to studies done in Thailand that found a sensitivity of platelet count <150,000/*μ*l for diagnosing malaria to be 85%, specificity 85%, negative predictive value 97%, and the positive predictive value 48% [31] and in Liberia that reported 80.11% sensitivity, 81.36% specificity, 63.87% positive predictive value, and 90.86% negative predictive value [32]. Our results also comparable to the findings of Arshad AR [21] and Tangvarasittichai O et al. [17].

## 5. Conclusion

Malaria is still among the major public health problems in the country. The prevalence of thrombocytopenia was significantly higher among malaria patients than malaria negative acute febrile patients in Ataye Hospital. Thrombocytopenia is a good discriminatory test for the presence or absence of malaria with 79.5% sensitivity and 86.3% specificity. Therefore, this may be used in addition to the clinical and microscopic parameters to heighten the suspicion of malaria.

## Figures and Tables

**Table 1:** Sociodemographic and clinical characteristics of study participants (*n* = 423).

Variables	Category	Number (%)
Age in years	≤14	60 (14.2)
	15–24	143 (33.8)
	25–34	119 (28.1)
	35–44	72 (17.0)
	≥45	29 (6.9)

Sex	Male	191 (45.2)
	Female	232 (54.8)

Residence	Urban	199 (47.0)
	Rural	224 (53.0)

Marital status	Single	195 (46.1)
	Married	222 (52.5)
	Widowed	6 (1.4)

Educational status	Illiterate	105 (24.8)
	Read and write	39 (9.2)
	Primary	87 (20.6)
	Secondary	96 (22.7)
	College and above	96 (22.7)

Occupational status	Student	172 (40.7)
	Government employee	46 (10.9)
	House wife	109 (25.8)
	Farmer	40 (9.5)
	Private employee	56 (13.2)

Malaria status	Positive	73 (17.3)
	Negative	350 (82.7)

Thrombocytopenia status	Thrombocytopenic	106 (25.1)
	Nonthrombocytopenic	317 (74.9)

Thrombocytopenia severity	Severe	9 (2.1)
	Moderate	43 (10.2)
	Mild	54 (12.8)
	Nonthrombocytopenia	317 (74.9)

**Table 2:** Characteristics of acute febrile patients with and without malaria infection.

Variables	Category	Malaria status		*P* value^∗^
		Positive *n* (%)	Negative *n* (%)	
Age in years	≤14	11 (18.3%)	49 (81.7%)	0.077
	15–24	33 (23.1%)	110 (76.9%)	
	25–34	17 (14.3%)	102 (85.7%)	
	35–44	6 (8.3%)	66 (91.7%)	
	≥45	6 (20.7%)	23 (79.3)	
Sex	Male	28 (14.7%)	163 (85.3%)	0.249
	Female	45 (19.4%)	187 (80.6%)	
Residence	Urban	22 (11.1%)	177 (88.9%)	0.002
	Rural	51 (22.8%)	173 (77.2%)	
Marital status	Single	41 (21.0%)	154 (79.0%)	0.163
	Married	31 (14.0%)	191 (86.0%)	
	Widowed	1 (16.7%)	5 (83.3%)	
Educational status	Illiterate	17 (16.2%)	88 (83.8%)	0.319
	Read and write	4 (10.3%)	35 (89.7%)	
	Primary	14 (16.1%)	73 (83.9%)	
	Secondary	23 (24.0%)	73 (76.0%)	
	College and above	15 (15.6%)	81 (84.4%)	
Occupational status	Student	39 (22.7%)	133 (77.3%)	0.066
	Government employee	9 (19.6%)	37 (80.4%)	
	House wife	15 (13.8%)	94 (86.2%)	
	Farmer	6 (15.0%)	34 (85.0%)	
	Private employee	4 (7.1%)	52 (92.9%)	
Thrombocytopenia status	Thrombocytopenic	58 (79.5%)	48 (13.7%)	<0.001
	Nonthrombocytopenic	15 (20.5%)	302 (86.3%)	
Thrombocytopenia severity	Severe	9 (12.3%)	0 (0.0%)	<0.001
	Moderate	29 (39.7%)	14 (4%)	
	Mild	20 (27.4%)	34 (9.7%)	
	Nonthrombocytopenia	15 (20.5%)	302 (86.3%)	
Platelet count		119.86 ± 75.549	263.28 ± 82.17	<0.001

**Table 3:** Diagnostic values of platelet count to predict malaria in acute febrile patients.

Parameter	Thrombocytopenia (PLT count <150,000 cells/*µ*l)
Sensitivity	79.5%
Specificity	86.3%
Positive predictive value	54.7%
Negative predictive value	95.3%
Area under the curve (AUC) (95% CI)	0.893 (0.847–0.938)

PLT = platelet count; CI = confidence interval.

## Data Availability

The authors confirm that all data underlying the findings are fully available without restriction. All relevant data are within the manuscript.

## References

[1] Chesbrough M. (2002). *District Laboratory Practice in Tropical Countries (Part 1)*.

[2] WHO (2019). *World Malaria Report 2019*.

[3] FDRE (2010). *National Strategic Plan for Malaria Prevention, Control and Elimination in Ethiopia 2011–2015*.

[4] Girum T., Shumbej T., Shewangizaw M. (2019). Burden of malaria in Ethiopia, 2000-2016: findings from the global health estimates 2016. Tropical diseases. *Travel Medicine and Vaccines*.

[5] EPHI (2016). *Ethiopia National Malaria Indicator Survey 2015. Federal Democratic Republic of Ethiopia Ministry of Health Addid Ababa, Ethiopia*.

[6] Wickramasinghe S. N., Abdalla S. H. (2000). Blood and bone marrow changes in malaria. *Best Practice & Research Clinical Haematology*.

[7] Sakzabre D., Asiamah E. A., Akorsu E. E. (2020). Haematological profile of adults with malaria parasitaemia visiting the volta regional hospital, Ghana. *Advances in Hematology*.

[8] Erhart L. M., Meshnick S. R., Buathong N. (2004). Hematologic and clinical indices of malaria in a semi-immune population of western Thailand. *The American Journal of Tropical Medicine and Hygiene*.

[9] Awoke N., Arota A. (2019). Profiles of hematological parameters in plasmodium falciparum and plasmodium vivax malaria patients attending tercha general hospital, Dawuro zone, south Ethiopia. *Infection and Drug Resistance*.

[10] Price R. N., Luxemburger C., Simpson J. A. (2001). Factors contributing to anemia after uncomplicated falciparum malaria. *The American Journal of Tropical Medicine and Hygiene*.

[11] Patel P., Patel M., Gamit B., Modi J., Kevadiya S., Padsala S. (2013). Thrombocytopenia in malaria: correlation with various prevalent species. *International Journal of Medical Science and Public Health*.

[12] Lampah D. A., Yeo T. W., Malloy M. (2015). Severe malarial thrombocytopenia: a risk factor for mortality in Papua, Indonesia. *The Journal of Infectious Diseases*.

[13] Srivastava K., Sharma M., Mitchell W. B. (2017). Malaria and thrombopoiesis: a possible mechanism for the malarial thrombocytopenia. *Journal of Immunology, Infection & Inflammatory Diseases*.

[14] McMorran B. J. (2019). Immune role of platelets in malaria. *ISBT Science Series*.

[15] Foote S., Burgio G., McMorran B. (2017). *Platelets in Malarial Infection: Protective or Pathological? Platelets in Thrombotic and Non-thrombotic Disorders*.

[16] Lacerda M. V. G., Mourão M. P. G., Coelho H. C. C., Santos J. B. (2011). Thrombocytopenia in malaria: who cares?,. *Memórias Do Instituto Oswaldo Cruz*.

[17] Tangvarasittichai O., Srikong M., Tangvarasittichai S. (2016). Platelet count and platelet indices used as potential markers for first malaria infection diagnosis. *International Journal*.

[18] Jairajpuri Z. S., Rana S., Hassan M. J., Nabi F., Jetley S. (2014). An analysis of hematological parameters as a diagnostic test for malaria in patients with acute febrile illness: an institutional experience. *Oman Medical Journal*.

[19] Khan S. J., Abbass Y., Marwat M. A. (2012). Thrombocytopenia as an indicator of malaria in adult population. *Malaria Research and Treatment*.

[20] Mikre K., Zerdo Z. (2016). Thrombocytopenia as marker for the diagnosis of malaria among malaria suspected patients in Arba Minch health center, Gamo Gofa zone, southern Ethiopia: a cross-sectional study. *African Journal of Science and Research*.

[21] Arshad A. R. (2015). Thrombocytopenia in malaria: can platelet counts differentiate malaria from other infections. *Journal of the College of Physicians and Surgeons Pakistan*.

[22] Feleke S. M., Animut A., Belay M. (2015). Prevalence of malaria among acute febrile patients clinically suspected of having malaria in the Zeway health center, Ethiopia. *Japanese Journal of Infectious Diseases*.

[23] Teklemariam A., Alemseged M., Adugna S. (2018). Malaria-intestinal helminthes co-infection among patients in Wolkite health center and Attat hospital, Gurage zone, southern Ethiopia. *Journal of Parasitology and Vector Biology*.

[24] Barkat H., Abd Alla A. B., Galander A., Salah T., Elfaki T., Nasir A. (2019). Prevalence of malaria and quantification of cytokine levels during infection in East Nile locality, Khartoum State: a cross-sectional study. *F1000Research*.

[25] Githinji S., Noor A. M., Malinga J. (2016). A national health facility survey of malaria infection among febrile patients in Kenya, 2014. *Malaria Journal*.

[26] Karunamoorthi K., Bekele M. (2009). Prevalence of malaria from peripheral blood smears examination: a 1-year retrospective study from the Serbo Health Center, Kersa Woreda, Ethiopia. *Journal of Infection and Public Health*.

[27] Igiri B. E., Paul C., Iquo B., Ofonime M., Okoduwa S. I. R., Gabriel C. (2018). Prevalence of malaria and available practice for its prevention among patients with febrile illness attending Ahmadu Bello University teaching hospital, Zaria, Nigeria. *Public Health and Preventive Medicine*.

[28] Feleke D. G., Gebretsadik D., Gebreweld A. (2018). Analysis of the trend of malaria prevalence in Ataye, North Shoa, Ethiopia between 2013 and 2017. *Malaria Journal*.

[29] Ullah H., Khan M. I. U., Suleman S. K. (2019). Prevalence of malaria infection in district Dir lower, Pakistan. *Punjab University Journal of Zoology*.

[30] Tesfaye S., Belyhun Y., Teklu T., Medhin G., Mengesha T., Petros B. (2012). Malaria pattern observed in the highland fringe of Butajira, Southern Ethiopia: a ten-year retrospective analysis from parasitological and metrological data. *MWJ*.

[31] Kotepui M., Phunphuech B., Phiwklam N., Chupeerach C., Duangmano S. (2014). Effect of malarial infection on haematological parameters in population near Thailand-Myanmar border. *Malaria Journal*.

[32] Bajubair M. A., Solwi A. A., Ghanem Y. (2005). Thrombocytopenia: a predictor of malaria among febrile patients in Liberia. *Infectious Diseases Journal of Pakistan*.

[33] Shaikh Q. H., Ahmad S. M., Abbasi A., Malik S. A., Sahito A. A., Munir S. (2009). Thrombocytopenia in malaria. *Journal of the College of Physicians and Surgeons–Pakistan*.

[34] Kassa D., Petros B., Messele T., Admassu A., Adugna F., Wolday D. (2005). Parasito-haematological features of acute Plasmodium falciparum and *P. vivax* malaria patients with and without HIV co-infection at Wonji Sugar Estate, Ethiopia. *Ethiopian Journal of Health Development*.

[35] Aktas G., Duman T., Atak B. (2020). Irritable bowel syndrome is associated with novel inflammatory markers derived from hemogram parameters. *Family Medicine & Primary Care Review*.

[36] Sit M., Aktas G., Ozer B. (2019). Mean platelet volume: an overlooked herald of malignant thyroid nodules. *Acta Clinica Croatica*.

[37] Hekimsoy Z., Payzin B., Örnek T., Kandoğan G. (2004). Mean platelet volume in type 2 diabetic patients. *Journal of Diabetes and Its Complications*.

[38] Sincer I., Gunes Y., Mansiroglu A. K., Aktas G. (2019). Differential value of eosinophil count in acute coronary syndrome among elderly patients. *The Aging Male*.

